# Temporally Regulated Traffic of HuR and Its Associated ARE-Containing mRNAs from the Chromatoid Body to Polysomes during Mouse Spermatogenesis

**DOI:** 10.1371/journal.pone.0004900

**Published:** 2009-03-31

**Authors:** Mai Nguyen Chi, Frédéric Chalmel, Eric Agius, Nathalie Vanzo, Khalid S. A. Khabar, Bernard Jégou, Dominique Morello

**Affiliations:** 1 CBD, CNRS UMR5547, IFR 109, Université Paul Sabatier, Toulouse, France; 2 Inserm, U625, Rennes, France; 3 Université Rennes I, Campus de Beaulieu, IFR-140, GERHM, Rennes, France; 4 Program in Biomolecular Research, King Faisal Specialist Hospital and Research Center, Riyadh, Saudi Arabia; Victor Chang Cardiac Research Institute (VCCRI), Australia

## Abstract

**Background:**

In mammals, a temporal disconnection between mRNA transcription and protein synthesis occurs during late steps of germ cell differentiation, in contrast to most somatic tissues where transcription and translation are closely linked. Indeed, during late stages of spermatogenesis, protein synthesis relies on the appropriate storage of translationally inactive mRNAs in transcriptionally silent spermatids. The factors and cellular compartments regulating mRNA storage and the timing of their translation are still poorly understood. The chromatoid body (CB), that shares components with the P. bodies found in somatic cells, has recently been proposed to be a site of mRNA processing. Here, we describe a new component of the CB, the RNA binding protein HuR, known in somatic cells to control the stability/translation of AU-rich containing mRNAs (ARE-mRNAs).

**Methodology/Principal Findings:**

Using a combination of cell imagery and sucrose gradient fractionation, we show that HuR localization is highly dynamic during spermatid differentiation. First, in early round spermatids, HuR colocalizes with the Mouse Vasa Homolog, MVH, a marker of the CB. As spermatids differentiate, HuR exits the CB and concomitantly associates with polysomes. Using computational analyses, we identified two testis ARE-containing mRNAs, *Brd2* and *GCNF* that are bound by HuR and MVH. We show that these target ARE-mRNAs follow HuR trafficking, accumulating successively in the CB, where they are translationally silent, and in polysomes during spermatid differentiation.

**Conclusions/Significance:**

Our results reveal a temporal regulation of HuR trafficking together with its target mRNAs from the CB to polysomes as spermatids differentiate. They strongly suggest that through the transport of ARE-mRNAs from the CB to polysomes, HuR controls the appropriate timing of ARE-mRNA translation. HuR might represent a major post-transcriptional regulator, by promoting mRNA storage and then translation, during male germ cell differentiation.

## Introduction

Spermatogenesis is a highly regulated process whereby the spermatogonial stem cells at the basal side of the seminiferous tubules divide and differentiate to give rise ultimately to spermatozoa. Once meiosis has taken place in spermatocytes, the newly formed haploid round spermatids will elongate and differentiate to spermatozoa by a process referred to as spermiogenesis. A remarkable event occurs during spermiogenesis, long before spermatids complete their differentiation into spermatozoa: histones are replaced by protamines, causing the compaction of the chromatin and a concomitant cessation of transcription [1], [2], whilst proteins continue to be made. Thus, in contrast to most somatic tissues, where transcription and translation are concomitant, mRNA transcription and protein synthesis are temporally disconnected in the male germ cells. Consequently, late-stage specific protein synthesis relies on the appropriate storage of translationally inactive mRNAs in transcriptionally silent germ cells [3]. Recent microarray analysis combined with sucrose gradient experiments were used to monitor mRNA movement between ribonucleoproteins (RNPs) and polysomes during germ cell differentiation [4]. This study showed that many mRNAs shift from the mRNPs where they are silent to polysomes where they are translated, late in spermatogenesis [4]. Among them, many encode RNA-binding proteins (RBPs) [4], arguing that regulated mRNA storage, stabilization and translation are needed to ensure stage-specific protein synthesis.

The discovery that mRNPs are localized in various discrete cytoplasmic granules and cycle between different subcellular compartments has opened up new areas of research on mRNA fate [5]. In somatic cells, mRNA storage/decay takes place in specific cytoplasmic granules, in particular, Stress Granules (SGs) and Processing Bodies (P. Bodies). While P. Bodies represent discrete mRNA decay/storage foci found in all cell types [6], [7], SGs are formed only under conditions of stress and function as dynamic mRNA sorting centres, acting as intermediates between polysomes and P. Bodies [8]. Neither P. Bodies nor SGs have been described in male germ cells. However, the germ cells of many organisms contain a perinuclear cytoplasmic cloud-like structure called “germ plasm” or “nuage”, characterized by the expression of VASA, an ATP-dependent DEAD-box RNA helicase, required for embryonic patterning, germ plasm assembly and germ cell functions in *Drosophila* [9]. In mammalian germ cells, the nuage counterpart is thought to be the chromatoid body (CB) [10], [11], identified by the expression of MVH, the Mouse VASA Homolog. In mice, MVH is exclusively expressed in germ cells [11] and required for spermatogenesis [12]. In addition to MVH, it was recently discovered that the CB contains polyadenylated RNAs [11], components of the microRNA pathway and various constituents of the P. Bodies [10]. Although CB functions remain to be fully apprehended, its enrichment in RNAs and RBPs has led to the proposal that the CB acts as a germ-cell-specific centre for mRNA storage and processing [10], [11].

In this paper, we describe a new component of the CB, HuR. This RBP belongs to the ELAV (Embryonic Lethal Abnormal Vision) family of proteins [13]. It was first identified in somatic cells for its ability to bind to AU-rich element (ARE) [14] and to increase the stability of ARE-containing mRNAs (ARE-mRNAs) [15]. Its function in germ cells has not yet been investigated. We demonstrate that HuR exhibits a remarkably dynamic cytoplasmic localization, trafficking between the CB and polysomes during spermatid differentiation. We have identified two of HuR's ARE-containing target mRNAs, *Brd2* and *GCNF*, which are highly expressed in spermatids, and show that traffic of HuR is accompanied by a sequential accumulation of its targets, first in the CB and then in polysomes, in which they become translationally active.

Our results strongly suggest that HuR translocation between the CB and polysomes controls the timing of ARE-mRNA translation during spermiogenesis.

## Results

### ARE-transcriptome is widely expressed in male germ cells

**Figure 1:**
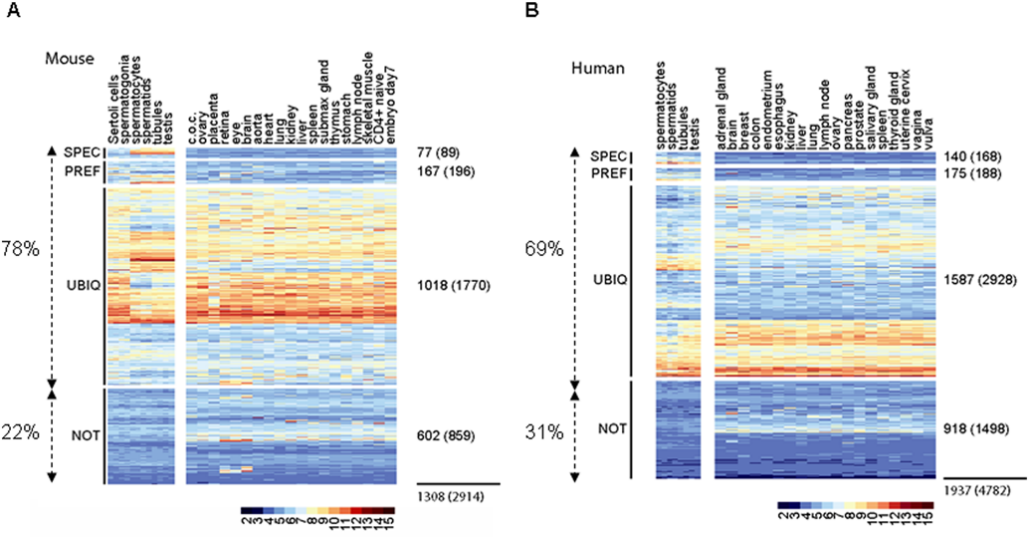
Analysis of testis ARE-transcriptome. A and B. Color-coded heat-maps showing expression patterns of 1308 or 1937 mouse and human genes (NCBI EntrezGenes identifiers), respectively, corresponding to the ARE-mRNAs from the ARED database in testicular somatic cells (Sertoli cells), germ cells (spermatogonia, pachytene spermatocytes, round spermatids), seminiferous tubules, testis and in 19 normal non testicular somatic tissues (as indicated). Columns and lines represent samples and Affymetrix probe set IDs (PSIDs), respectively. Log2-transformed intensity signals are colored according to the scale bars. Using the filtration methods described in [19], PSIDs are classified according to their specific (SPEC), preferential (PREF, with one exception in the 19 normal non-testicular samples), ubiquitous (UBI, expressed in more than two normal non-testicular samples) or lack (NOT, not detected in testicular samples) of expression in testis/germ cell samples (on the left) as compared to the 19 normal and healthy non-testicular samples. Background expression cutoff was set at 6.54 and 4.79 for mouse and human transcriptome analysis, respectively. Numbers of genes and PSIDs are indicated at the right of each class. Note that the lines between A and B heat-maps do not correspond to the same PSID.

Mammalian AREs are *cis*-elements located in the 3′ untranslated region (3′ UTR) of many mRNAs with a diverse functional repertoire [16], for which they ensure accurate transport, stability, storage and translation in somatic cells [17]. No data are yet available on ARE-mRNA expression during spermatogenesis. To gain insight into this question, we analyzed the expression of ARE-transcripts in male germ cells. We first listed all murine and human ARE-mRNAs from the ARED Organism database (ARED 3.0: http://brp.kfshrc.edu.sa/ARED/ and [18]. They were associated with 1308 mouse and 1937 human non-redundant NCBI EntrezGene identifiers as well as with 2914 mouse and 4724 human Affymetrix probe set identifiers from the Mouse430_2 and the HG-U133_Plus_2 GeneChips, respectively. Then, to estimate ARE-mRNA expression pattern during germ cell differentiation, we used previously assembled and preprocessed transcriptome data (RMA normalization) described in [19]. Concerning the mouse, this dataset includes one somatic testicular sample (Sertoli cells), three purified germ cell samples (spermatogonia, pachytene spermatocytes and round spermatids) as well as seminiferous tubules and whole testis whose RNAs were hybridized to Mouse430_2 Affymetrix GeneChips (Figure 1 and Tables S1 and S2). With respect to the human dataset, two purified germ cell samples (pachytene spermatocytes and round spermatids), seminiferous tubules and whole testis were hybridized to HG-U133_Plus_2 GeneChips (Tables S1 and S2).

Then, to evaluate the tissue specificity of a given ARE-mRNA, we compared the level of expression of its corresponding probe sets in testicular samples with 19 healthy murine or human non-testicular tissue samples (detailed in Figure 1 and Table S2). Most mouse ARE-mRNAs (78%) were detected in testis, some being exclusively (6%, no exception) or preferentially (13%, one exception in the normal non-testicular tissues) expressed in this tissue (Figure 1A and Table S2). Almost one third of these specifically or preferentially expressed transcripts were only detected in meiotic (spermatocytes) and post-meiotic (spermatids) germ cells, and were not found in somatic (Sertoli) or mitotic (spermatogonia) testicular cells. Similar results were observed when the ARE-transcriptome of human germ cells was analyzed (Figure 1B and [16]). ARE-mRNAs were enriched for several Gene Ontology (GO) terms, including reproduction (p value <0.001). In addition, the search for ARE-transcripts annotated for “spermatogenesis” GO term revealed seventeen transcripts with assigned functions, the main characteristics of which are described in Table 1. The KO of fourteen of the corresponding genes has been generated. Five are embryonic lethal. Of the remaining 9, 8 were shown to be involved in male germ cell differentiation (Table 1). All together, our data strongly suggest that ARE-mRNAs play important roles during spermatogenesis.

**Table 1:** Principal characteristics of ARE-containing mRNAs expressed in spermatogenesis.

Gene	ARE Class	Chr	Expression	Loc.	Molecular function term	KO	Defects	Ref.
*Brd2*	4	17	Whole testis	N/c	Serine/threonine kinase	No		
*Dmc1*	5	15	Whole testis	N	Recombinase activity/Meiotic rec.	Yes	Spermatogenesis	[44], [45]
*Fshb*	3	2	Whole testis	S	Follicule stimulating horm. Activity	Yes	small testis	[46]
*Gmcl1/Gcl1*	5	6	Meiotic	N	Protein binding/nuclear morphol.	Yes	Spermatogenesis	[47]
*Golga3/Mea2*	5	5	Whole testis	C	Protein binding/Golgi apparatus	Yes	Spermatogenesis	[48]
*Gpr64/He6*	5	X	Sertoli	Mb	G-protein coupled receptor activity	Yes	Spermatogenesis	[49]
*Hsf2*	5	10	Whole testis	N/C	Transcription regulator	Yes	small testis	[50]
*Nr6A1/Gcnf*	5	2	Whole testis	N	Transcription inhibitor	Yes	embryonic lethal	[51]
*Odf2*	3	2	Whole testis	C	Cytoskeleton	Yes	Preimplantation lethality	[52]
*Prok 2*	5	6	Whole testis	Mb	G-protein coupled receptor bind.	Yes	Spermatogenesis	[53]
*Qk*	5	17	Mitotic	N/C	RNA binding protein	Yes	embryonic lethal	[54]
*Rgs2*	5	1	Mitotic	N	Regulator of G-protein signalling	Yes	No	[55]
*Slc2a3/Glut3*	3	6	Meiotic	Mb	Glucose transporter	Yes	embryonic lethal	[56]
*Spata 6*	5	4	Post-Meiotic	Mb	unknown	No		
*Spo11*	5	2	Meiotic	N	DNA topoisomerase/Meiotic rec.	Yes	Spermatogenesis	[57], [58]
*Tcp11*	5	17	Whole testis	Mb	?	No		
*Ufd1*	5	16	Meiotic	C	DNA binding/ubiquitin cycle	Yes	embryonic lethal	[60]

For each given gene, we have indicated the class of ARE that its 3′ UTR contains, according to http://brp.kfshrc.edu.sa/ARED, their chromosomal localization (Chr) and the site of expression of the corresponding protein (N: nucleus, C: cytoplasm, Mb: membrane, S: secreted). Expression indicates the type of cells where the level of mRNA is the most important (meiotic; spermatocytes, post-meiotic: spermatids, mitotic: Sertoli and/or spermatogonia; whole testis indicates that mRNA is expressed uniformly in the different cell types). When known, the function of the protein is also indicated. Defects observed when a KO has been performed (Yes) are mentioned. No indicates that no corresponding KO was obtained.

### HuR expression is tightly regulated during germ cell differentiation

**Figure 2:**
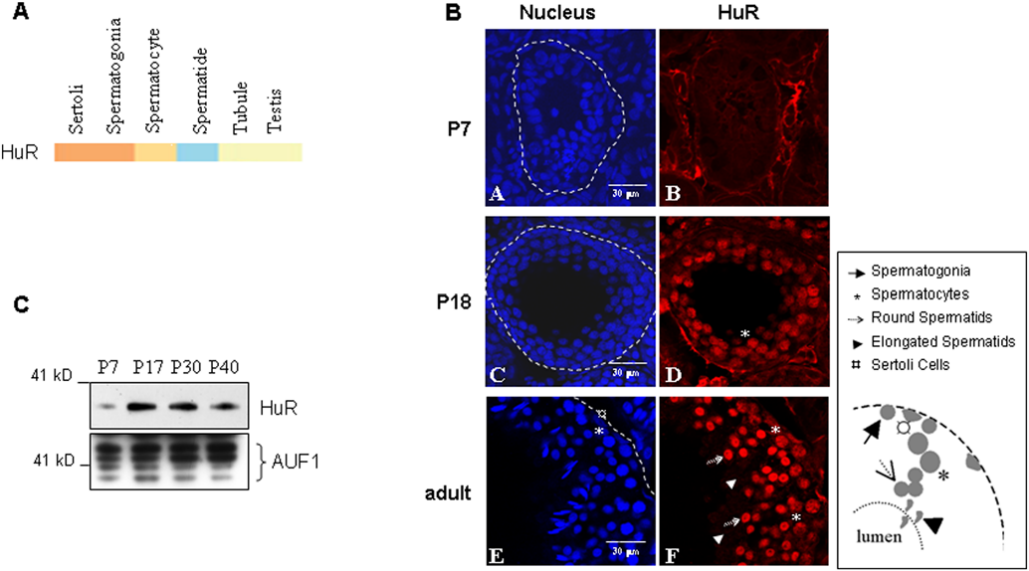
Analysis of HuR expression during testis ontogenesis. A. Heat map representing *HuR* mRNA expression in germ cells, tubules and testis [19], showing its higher expression in early stages of differentiation. Color-code as in Figure 1. B. Sections (approximately 60 µm) of seminiferous tubules at the indicated ages were stained with monoclonal anti-HuR antibodies and analyzed by confocal immunofluorescence microscopy to reveal HuR (red, B, D, F). Nuclei were stained with chromomycin (blue, A, C, E). Scale bars, as indicated. The drawings represent spermatogenesis in adult seminiferous tubules. Differentiation proceeds from the basal membrane to the lumen of the tubule, each cell type being represented by the symbols described above. C. HuR expression at various stages of germ cell differentiation was analysed by Western blot. The membrane was subsequently stained with anti-AUF1 antibody to control protein loading.

The regulation of ARE-mRNA expression in testis might imply interaction with ARE-binding proteins. Among these, we focused on HuR because we previously showed that its overexpression in transgenic testis led to impaired spermatogenesis [20]. We examined HuR expression both at mRNA and protein levels in spermatogenic cells, using mouse testes from P7, P18 and P28 pre-pubertal or adult males. In the mouse, for the first few days after birth, the seminiferous tubules contain gonocytes, spermatogonia and Sertoli cells. Spermatocytes appear at day 10 and haploid spermatids and spermatozoa after day 20 and 35, respectively [21]. HuR mRNA is initially expressed in spermatogonia and the somatic Sertoli cells and its expression subsequently decreases, as shown by microarray transcriptome database [19] and Figure 2A). By contrast, using immunohistochemical staining of sections of pre-pubertal and adult testes, we observed that HuR protein is absent from spermatogonia (Figure 2B, panel B) and is only weakly expressed in spermatocytes (P18 and adult, Figure 2B, panels D and F). Later, its expression peaks in round spermatids and becomes undetectable at later steps of spermatid differentiation (Figure 2B, panel F). That HuR is weakly expressed at an early stage of germ cell differentiation was further confirmed by Western blot (Figure 2C). Thus, HuR is not expressed in all germ cells and is abundant at the beginning of haploid phase.

### HuR accumulates in the chromatoid body of early round spermatids

**Figure 3:**
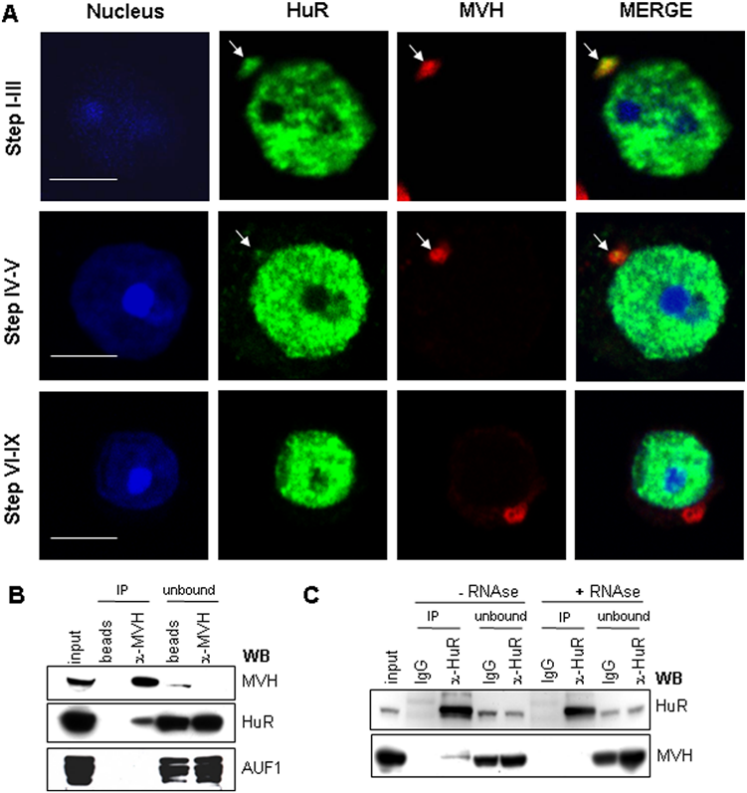
HuR and MVH colocalize within the chromatoid body. A. Round spermatids at the indicated steps of differentiation were prepared from tubule squashes of P40 testis. Cells were co-stained with anti-HuR (green) and anti-MVH (red) antibodies and were observed by confocal microscopy. Both proteins colocalize within the CB of early spermatids (yellow in merge pictures). No colocalization is seen when spermatids mature (step-VI-IX). Scale bar: 5 µm. B. Western blot (WB) analysis of MVH immunoprecipitate (IP) from cytoplasmic extracts of P40 testis shows that MVH and HuR co-immunoprecipitate. Input corresponds to 1/20 of the fraction immunoprecipitated with anti-MVH or control IgG (beads). One-twentieth of the unbound material was loaded on the gel in parallel. The membrane was sequentially incubated with anti-MVH, anti-HuR or anti-AUF1 antibodies to analyze the specificity of the MVH-HuR interaction. C. The same experiment was performed with HuR IP, untreated or treated with RNase. The membrane was sequentially incubated with anti-HuR and -MVH antibodies.

Interestingly, besides its prominent expression in the nucleus of early round spermatids, HuR is also detected in a perinuclear cytoplasmic structure that contains MVH (Figure 3A, step I–III) and polyadenylated mRNAs (Figure S1) and thus corresponds to the CB. Association between HuR and MVH was confirmed by reciprocal co-immunoprecipitation experiments (coIP) using anti-MVH (Figure 3B) or anti-HuR (Figure 3C) antibodies on cytoplasmic extracts of young adult male germ cells. These experiments also revealed that HuR-MVH association is specific and RNA-dependent, since IPs do not contain AUF1, another ARE-binding protein [22] (Figure 3B), and RNAse treatment destroys HuR-MVH interaction (Figure 3C).

To further document HuR localization in the CB of spermatids, we micro-dissected segments of adult seminiferous tubules corresponding to different stages of differentiation [23], [24]. A minimum of 100 cells at a given stage of maturation was analyzed for HuR and MVH staining. We observed the colocalization of HuR and MVH in the CB of nearly all (95%) early round spermatids (step I–III) and of 69% of step IV–V spermatids (Figure 3A). Remarkably, HuR is no longer visible in the MVH-stained CBs of more advanced spermatids (Figure 3A, VI–IX). Taken together, our results indicate that HuR expression during spermatogenesis is dynamic and transiently localizes to the CB of early round spermatids.

### Association of HuR with polysomes increases with age

**Figure 4:**
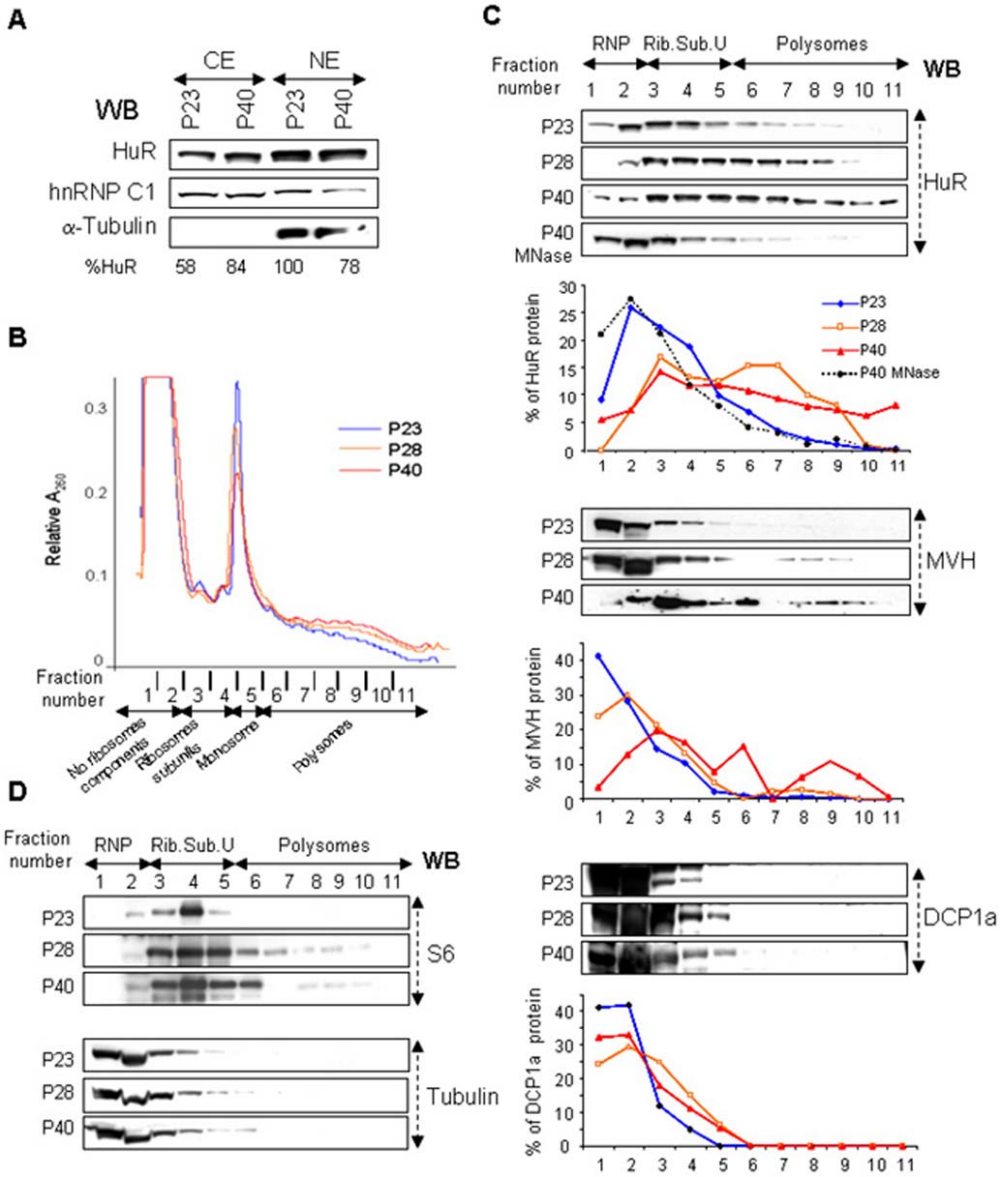
HuR associates with polysomes in late spermatids. A. Comparative analysis of HuR expression between P23 and P40 germ cells. The level of HuR expression in cytoplasmic (CE) and nuclear (NE) extracts was analyzed by Western blot using anti-HuR antibody. Levels of tubulin and hnRNP C expression reveal that equivalent amount of cytoplasmic (50 µg) and nuclear (10 µg) proteins were used in P23 and P40 extracts, respectively. Signals observed with hnRNPC1 in NE preparations indicate that the nuclear preparations were contaminated with cytoplasmic components. B, C, D. Comparative sucrose gradient analysis of cytoplasmic extracts from P23, P28 and P40 testes. Cytoplasmic extracts were prepared from a pool of 8–10 testes of the indicated age and loaded on a 15–50% sucrose density gradient. Eleven fractions were collected from each gradient and absorbance tracing at 260 nm was measured. For a given tracing, each point is represented as a percentage of total recovered absorbance. Tracings were superimposed to show that translation efficiency increases with age. The data shown are representative of two independent experiments. The direction of sedimentation (top to bottom of the gradient) and the components of the translational machinery in each fraction are indicated: no ribosome component, corresponding to RNPs, small and large ribosomal sub-units and monosome (Rib. Sub.U) and polysomes. Proteins were extracted from each fraction and expression profiles of control ribosomal sub-unit S6 and α-tubulin (C), HuR, MVH and Dcp1a (D) in P23, P28 and P40 cytoplasmic germ cell extracts were analyzed by Western blot. The graphs represent the percentage of the intensity of the signal in a given fraction expressed relative to the sum of the intensities found in the 11 fractions, considered as 100%. HuR sedimentation with polysomes was tested by treating P40 cytoplasmic extract with *Staphylococcus aureus* nuclease (MNase), which cleaves mRNA between polysomes. HuR initially sedimenting in both RNPs and polysomes containing fractions switches to low density fractions after MNase treatment.

To determine whether the disappearance of HuR from the CB is due to its cytoplasmic degradation during spermatid differentiation, we prepared cytoplasmic extracts (CE) of prepubertal testes (P23), where the early round spermatid stage (I–III) is reached, and young adult testes (P40), which contain all stages of spermatid differentiation, including late round and elongated spermatids. We observed that the level of HuR expression does not decrease but even slightly increases in P40 cytoplasmic extract (Figure 4A), indicating that HuR is not degraded in the cytoplasm of advanced spermatids. Concomitantly, its nuclear expression is similar in the early and late nuclear extractions (NE, Figure 4A). Al together, these results show that HuR which is mainly concentrated in the CB of early spermatids is not degraded while spermatids mature but accumulates in another cytoplasmic compartment.

To find out whether HuR associates with polysomes, we fractionated germ cell cytoplasmic extracts on sucrose gradients. In addition to P23 and P40, we used P28 testis extract, which is enriched in late round spermatids (Figure 4B). As shown by Western blot analysis, in the early phase of spermatid maturation (P23), HuR is predominantly found with low-density fractions, corresponding to RNPs, and ribosomal sub-units (Figure 4C). According to Grivna and collaborators, the CB sediments in the RNP fractions, as two of its components, GW182 and MVH, distribute in RNPs of P24 sucrose gradients [25]. Using DCP1a as another recognized marker of the CB [26], we confirmed that the CB accumulates in RNPs at the early stage of differentiation (P23) (Figure 4C). Combined with data presented above, this result strongly suggests that the signal of HuR in low density fractions corresponds to CB-associated HuR. Remarkably, at later stages of spermatid maturation (P28 and P40), while DCP1a stays concentrated in RNP fractions, a portion of HuR and MVH sediments into denser regions of the gradients (Figure 4C), suggesting that a fraction of these two RNA binding proteins leaves the CB and associates with polysomes while differentiation proceeds. To validate this hypothesis, we treated P40 cell extracts before sedimentation with micrococcal nuclease, which dissociate polysomes. This treatment resulted in a shift of HuR and MVH (not shown) to low density fractions (Figure 4C, MNase and data not shown), indicating that these two RNA-binding proteins associate with polysomes at late stages of spermatid differentiation.

Comparison of RNA absorbance profiles between P23, P28 and P40 extracts shows a slight but reproducible increase in the amount of polysomes with increasing age, accompanied by a reduction in the monosome-containing fraction (Figure 4B). We also observed a shift of the small ribosomal sub-unit protein S6 from fractions 3 and 4 in the P23 gradient to denser fractions in the P28 and P40 gradients, while the content of tubulin, taken as an internal control, in a given fraction is similar in the three gradients (Figure 4D). All together, our results show that HuR translocates from the CB to polysomes while spermatids mature and that HuR and MVH association with polysomes correlates with a weak but significant increase of mRNA translation in late germ cells.

### HuR-target mRNAs are dynamically localized during spermatid differentiation

The transfer of HuR from the CB to polysomes suggests that HuR may regulate storage and translation of mRNAs during spermiogenesis. Testing this hypothesis requires identification of mRNAs present in the CB. Unfortunately, such information is not yet available. In an attempt to characterize HuR-target mRNAs in the CB, we first searched for ARE-mRNAs associated with HuR in the testis. For this purpose, we used HuR transgenic mice (HuR^tg^), which overexpress HuR protein specifically in their testes [20] and compared ARE-transcriptomes of HuR^tg^ testes with those of wild-type (WT) testes, using the ARE-cDNA microarray system [27]. Among other mis-regulated ARE-mRNAs (our unpublished data), we selected *Germ Cell Nuclear Factor* (*GCNF/Nr6A1*) and *Bromodomain 2* (*Brd2*) mRNAs for further analysis because 1) they are expressed in spermatids (Figure S2A and [28], [29], 2) they were shown to be involved in spermatogenesis (Table 1 and [28], [29]) and 3) their 3′ UTR contains AREs conserved in rat and human (Figure S2B).

**Figure 5:**
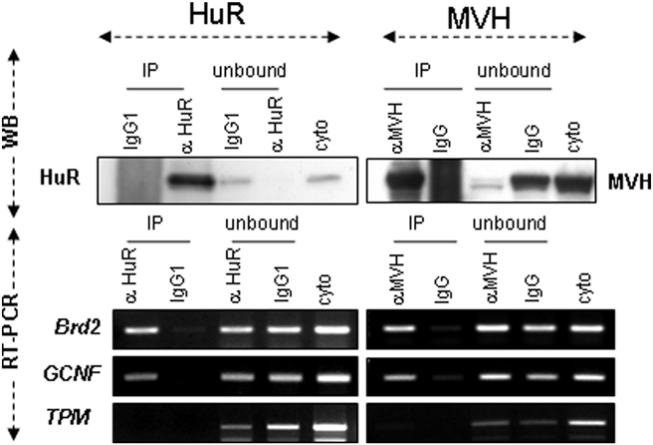
*Brd2* and *GCNF* mRNAs are bound by HuR and MVH. Cytoplasmic germ cell extracts from P40 testes were incubated with anti-HuR or anti-MVH antibodies to immunoprecipitate HuR or MVH and their associated mRNAs. Proteins and RNAs were extracted. Levels of HuR and MVH in the immunoprecipitated (IP) or unbound materials (unbound) were analyzed by Western blot (WB). Levels of *Brd2*, *GCNF* and control *TPM1* mRNAs in each fraction (bound (IP), unbound and crude cytoplasmic extract: cyto) were analyzed by semi-quantitative RT-PCR.

Next, to ascertain whether *GCNF* and *Brd2* mRNAs are *bona fide* HuR-target mRNAs, we tested their ability to bind HuR protein. To this end, we performed RNA-IP experiments using adult cytoplasmic germ cell extracts and anti-HuR antibody (Figure 5). We found that *GCNF* and *Brd2* mRNAs, but not control *tropomyosin* (TPM1) mRNAs, specifically bind HuR. As HuR and MVH associate in the same mRNP complexes (Figure 3B, C), we also observed HuR-target mRNAs in MVH IPs (Figure 5).

**Figure 6:**
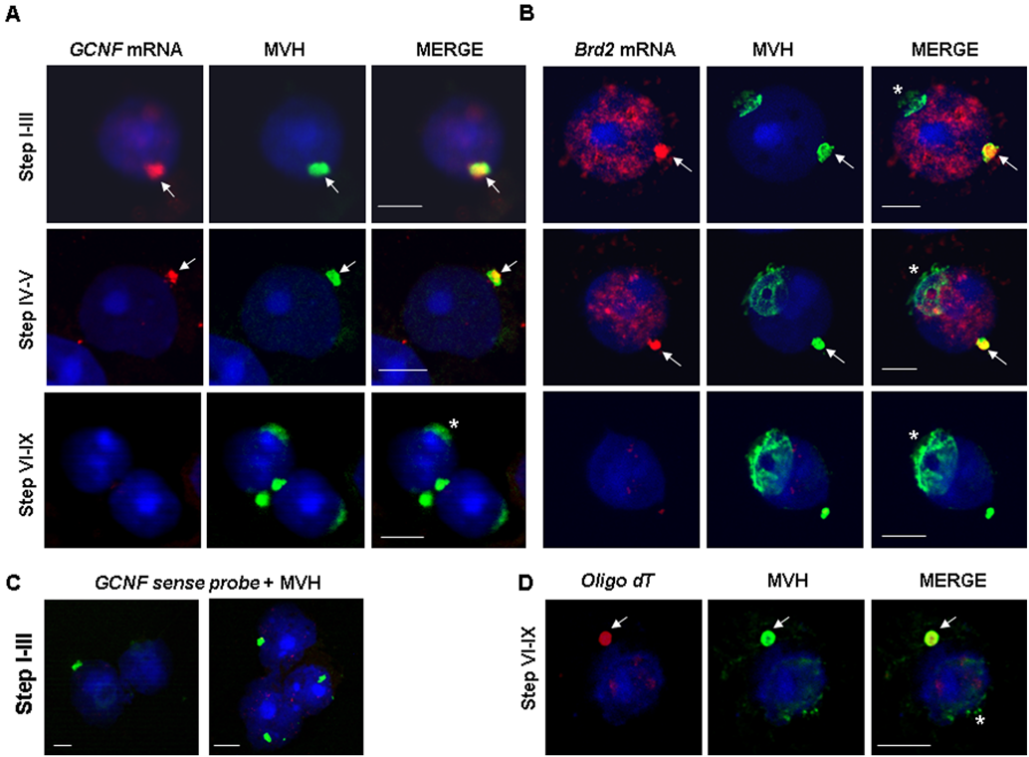
ARE-mRNAs transiently localized within the CB of spermatids. A, B. *In situ* hybridization and immunohistochemistry were performed on dried down slides from adult testis to study *GCNF* (A) and *Brd2* (B) mRNA localization (anti-sense probe: red). The CB is stained by anti-MVH antibody (green). Merge pictures show localization of *GCNF* and *Brd2* mRNAs in the CB (yellow+arrows) of early round spermatids (Step I–III and IV–V). No mRNA signal is observed in CBs once spermatids mature (steps VI–IX). Scale bar: 5 µm. Asterisk (*) denotes unspecific labeling of the acrosome with the anti-MVH antibody. Nuclear staining of early round spermatids with the *Brd2* probe most probably corresponds to *Brd2* neo-transcripts or transcripts synthesized during meiotic stages. C. *GCNF* sense probe was used on Step I–III spermatids to assess specificity of anti-sense probe localization in the CB. D. Oligo-dT and anti-MVH antibody were used simultaneously to study persistence of polyadenylated mRNAs in CBs of mature round spermatids (Step VI–IX).

We then determined whether *GCNF* and *Brd2* mRNAs localize within the CB. Combining *in situ* hybridization and immunofluorescence, we observed that both mRNAs colocalize with MVH (Figures 6A, B), showing that they do indeed accumulate in CBs of early spermatids (up to stage V), where HuR also resides (Figure 3A, Step I–III and Figures S3A, B). Absence of signal with the sense probe demonstrates the specificity of *GCNF* and *Brd2* mRNA detection in the CB (Figure 6C for *GCNF*; not shown for *Brd2*). At later stages of spermatid differentiation, *GCNF* and *Brd2* hybridization signals in CBs disappear (Figure 6A, B, Step VI–IX). As observed using oligo dT probe, polyadenylated mRNAs remain accumulated in the CB of late stage spermatids (Figure 6D, oligo dT). These data show that the temporally control disappearance of mRNAs from the CB is not a general process but applies to a restricted population of mRNAs, including those containing AREs.

**Figure 7:**
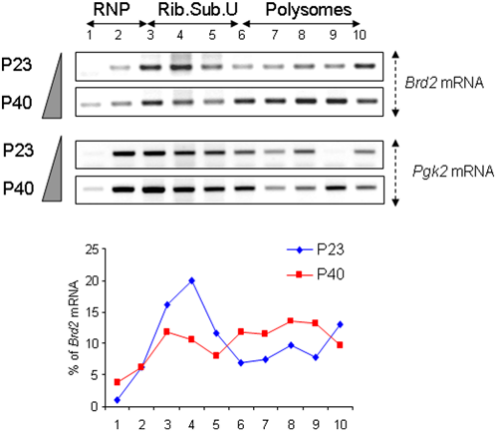
Increased association of *Brd2* mRNAs with polysomes in aged spermatids. Germ cell cytoplasmic extracts from a pool of P23 or P40 testes were fractionated on 15–50% sucrose density gradients. RNAs were extracted and expression of *Brd2* and control *Pgk2* mRNAs was analyzed by semi-quantitative RT-PCR. *Brd2* mRNAs partially shift from low density fractions at P23 to denser fractions at P40. In prepubertal and young adult testes, *Pgk2* mRNA is found in both mRNPs and polysomes. The graph below represents the percentage of the *Brd2* signal in a given fraction expressed relative to the sum of the intensities found in the 10 fractions, considered as 100%.

Disappearance of *Brd2* and *GCNF* mRNAs from CBs could correspond to their accumulation in polysomes in late spermatids. To test this hypothesis, we prepared RNAs from P23 and P40 sucrose gradients and analyzed by RT-PCR the content of *GCNF* and *Brd2* mRNAs in each fraction. Analysis of *GCNF* is hampered by the existence of two *GCNF* transcripts, differing in their 3′ UTR, whose expression partly overlaps during spermatogenesis [29] (Figure S2C). As shown in Figure 7, *Brd2* mRNAs shift from mRNPs to polysomes with increasing age. *Pgk2* mRNA, which does not contain an ARE, was used to control RNA quality. It accumulates both in mRNPs and HMW fractions, as previously observed [4], [29], [30] and its expression profile does not significantly change between P23 and P40. All together, these results show that two ARE-containing mRNAs, encoding important actors of male differentiation, exhibit a temporally regulated localization: initially concentrated within the CB of early round spermatids, they subsequently become associated with polysomes, concomitantly with HuR, at later stages of differentiation, as demonstrated for *Brd2*.

## Discussion

During spermatogenesis, in contrast to what is observed in most somatic cells, a large quantity of mRNAs accumulated during transcription are stored as mRNPs, which are subsequently assembled into polysomes [31]. The mechanisms underlying mRNA storage and regulated translation remain obscure. Based on the results presented here, showing the exquisitely regulated spatio-temporal expression of HuR and its target mRNAs, we propose that HuR is a key player in the post-transcriptional events required to store mRNAs and control their translation at post-meiotic stages.

**Figure 8:**
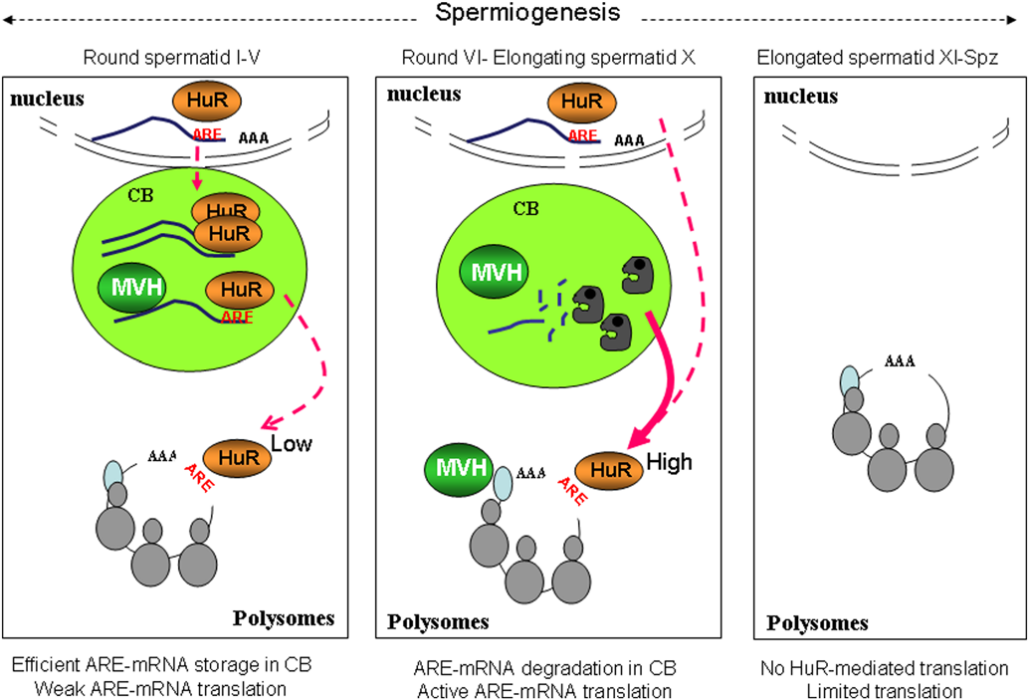
Model for HuR role during spermiogenesis. In early round spermatids, HuR, mainly located in the nucleus, is exported to the CB together with its associated target ARE-mRNAs. There, it participates in the storage of long-lived mRNAs and their protection from degradation. However the constant “feeding” of the CB with mRNAs could also favor a rapid shuttling of a small fraction of mRNAs to polysomes, resulting in translation of HuR-target mRNAs at a low level. After step V spermatids, HuR translocates from the CB to polysomes with its associated ARE-mRNAs, where, together with MVH, it contributes to their translation. Due to loss of HuR from the CB, the HuR-targets remaining in this structure are no longer protected from degradation and are consequently destroyed by degradation enzymes and miRNA-mediated processing. Subsequently, in elongated spermatids, transcription has ceased. Most mRNAs have been either degraded or translated at earlier stages of spermiogenesis. The pool of mRNAs remaining at these late stages is restricted to those, such as *protamine* mRNA, required for the ultimate steps of spermiogenesis. This model highlights the necessity for spermatids to concomitantly translate mRNAs and store them during the extended haploid differentiation period.

HuR, first detected at a low level in mid-pachytene primary spermatocytes, is massively expressed in spermatids. Its peak of expression at the early round spermatid stage (I–V) coincides with the second wave of transcription that occurs post-meiotically [11], [32] and generates many long-lived transcripts whose translation is delayed until later stages of spermiogenesis [4]. HuR predominantly localizes to the nucleus of early round spermatids, but is also found in their CB, a unique cloud-like structure, which, due to its frequent contacts with the spermatid nucleus from step II to step IV, is thought to be involved in the transport of RNAs from the nucleus to the cytoplasm [11]. Thus, it is possible that HuR associates with its target mRNAs in the nucleus of germ cells, as observed in somatic cells [33], [34] and, taking advantage of frequent contacts between nucleus and CBs at the beginning of spermatid differentiation [11], transports its associated mRNAs to the CB (Figure 8). This structure includes several enzymes involved in mRNA degradation, such as the decapping enzyme DCP1a, GW182 and MVH or components of the RNAi machinery [26]. The fact that the level of *Brd2* and *GCNF* mRNAs does not decrease as differentiation proceeds (not shown) indicates that HuR targets are not degraded in CBs of early round spermatids. Due to its mRNA-stabilizing activity, HuR could protect them from degradation in this structure. In addition, since CBs do not contain components of the translational machinery, accumulation of HuR targets in CBs constitutes an efficient means to prevent their premature translation [10]. HuR could also interact with the miRNAs that were recently shown to accumulate in the CB of haploid spermatids [35], thereby facilitating translational repression of its target mRNAs in the CB. As exemplified here for *GCNF* and *Brd2* mRNAs, the CB could be therefore a privileged platform for ARE-mRNA regulation.

Subsequently, from stage VI and onwards, HuR progressively exits the CBs of round spermatids and translocates from mRNPs to polysomes. Concomitantly, HuR-target mRNAs disappear from the CB and associate with polysomes, suggesting that HuR participates in their shuttling toward these structures. As a part of MVH is also found in polysomes and binds HuR-target mRNAs, our data do not exclude the contribution of MVH to ARE-mRNA trafficking. The association of HuR and MVH could favor increased translation of HuR targets in round late spermatids, since VASA is known to activate translation in *Drosophila* [36]. Relocalization of ARE-mRNAs from a translationally silent site towards polysomes could remove miRNA-mediated repression, a situation described in hepatocarcinoma cells, where HuR impairs miRNA-mediated repression of *Cat-1* mRNA in P. Bodies by relocalizing this mRNA to polysomes [37].

To summarize our data, we propose the model described in Figure 8 in which, through its temporal translocation from the nucleus to the CB and subsequently from the CB to polysomes, HuR controls the stage-dependent transport, storage and translation of its target mRNAs during spermiogenesis. HuR KO being embryonic lethal (Kontoyiannis, personal communication), germ-cell-specific inactivation of HuR will be required to unravel the sophisticated HuR-mediated post-transcriptional controls occurring in haploid spermatids.

The molecular mechanisms controlling HuR trafficking between different germ cell compartments remain to be characterized. HuR transit from the CB to polysomes could rely on post-translational modifications, such as phosphorylation or methylation, shown to be important in somatic cells for HuR translocation from nucleus to cytoplasm [15], [38]–[41]. Because HuR and MVH also interact through common mRNA-targets, it is also possible that HuR exit from CBs requires MVH or other germ-cell-specific components such as MIWI, the association of which with piRNAs and polysomes increases with age [25]. The potential role of MVH in driving HuR from the CB could be tested using *mvh*-deficient germ cells. However, an experiment of this sort will require production, *via* conditional knock-out, of *mvh* post-meiotic mutant germ cells, since *mvh* mutant mice arrest spermatogenesis before the formation of the CB [12].

Comparative analysis of ARE-repertoire [18] and germ cell transcriptome [19] revealed that numerous ARE-containing mRNAs are expressed in mouse and human germ cells. They represent 7% of the genes transcribed in testis. The two HuR-target mRNAs characterized in this study encode for important regulators of spermatogenesis. GCNF is a nuclear protein that acts a transcriptional repressor [42], [43]. *Brd2* mRNA encodes a bromodomain-containing protein that may be involved in chromatin remodeling during spermatogenesis [28]. In addition to *GCNF* and *Brd2* mRNAs, we identified 15 ARE-mRNAs expressed in testis. Of these 15 ARE-mRNAs, KO models for 5 lead to embryonic lethality and, thus are not informative. On the other hand, 8 of the 10 remaining genes lead to defects in germ cell differentiation and sterility (Table 1 and [44]–[59]. This strongly suggests that many ARE-containing mRNAs are essential for germ cell differentiation. Their conserved ARE sequences in mammals [18] also speaks for their key regulatory role during spermatogenesis. In addition, *GCNF*, *Gmcl1*, *Odf2*, *Ufd1*, *Spata6* and *Brd2* move between RNPs and polysomes [4], leaving open the hypothesis that ARE/ARE-binding protein interaction mediates their up- or down-translation.

In addition to ARE-mRNAs characterized by the presence of the WWWUAUUUAUWWW motif (http://brp.kfshrc.edu.sa/ARED), HuR can also bind to a 17- to 20-base-long RNA motif rich in uracils present in mRNAs expressed in the human colorectal carcinoma cell line RKO [60]. Therefore, HuR could co-ordinately regulate the expression of a range of mRNAs whose accurate translation is required for spermiogenesis to unfold correctly.

## Materials and Methods

### Purification of spermatogenic cells and tubule squashes; immunohistochemistry on testis sections and *in situ* hybridization on tubule squashes

Mice were maintained in accordance with institutional guidelines (CNRS). Isolated spermatogenic cells were prepared from decapsulated testes. Tubules were incubated in 5 ml phosphate-buffered saline (PBS) containing 1 mg/ml collagenase, with continuous agitation at 200 rpm at 34°C for 15 min. The dispersed seminiferous cords and cells were allowed to sediment for 10 min at 1200 rpm and the supernatant was removed. The pellet was resuspended in 5 ml 0.25% trypsin/EDTA and incubated under the same conditions as above. The resulting solution was supplemented with 5 ml fetal calf serum, then mixed and the cell suspension was filtered through nylon filters (70 µm mesh) and washed in 30 ml PBS. The cells were pelleted by centrifugation at 1500 g for 10 min.

Tubule squashes were prepared as described in [24] except that germ cells were not frozen, but directly fixed in 4% paraformaldehyde. To prepare the stage-specific dried-down slides, segments of seminiferous tubules were isolated [24] and transferred to 30 µl of 100 mM sucrose solution. Cells were released from the tubules by careful squeezing with a coverlip and were suspended by pipetting. The cell suspension was spread on a slide dipped in the fixing solution (2% paraformaldehyde, 0.05% Triton X-100) and the slides were dried for 1 h in the dark.

Sixty-µm sections of testis of various ages (P7, P18 and adult), treated as described by [20] were stained with monoclonal anti-HuR antibody (19F12, a generous gift from H. Furneaux). CBs in squashed cells were stained by polyclonal anti-MVH antibody (1/200 Abcam) or monoclonal anti-HuR antibody. Immunostaining was performed using a rhodamine-conjugated goat anti-mouse (Alexa 546, Molecular Probe) or anti-rabbit antibody (Alexa 488). Nuclei were labelled with either chromomycin A3 (Sigma) or TO-PRO3 (Molecular Probes). Images were obtained with a Leica SP2 confocal microscope equipped with helium-neon lasers and appropriate filter combinations.

*In situ* hybridization was performed on dried-down slides as described in [61], with some modifications. Cells were incubated with DIG-labelled RNA probes for 4 h at 45°C. CBs and RNAs were revealed concomitantly by incubating the cells with polyclonal anti-MVH (1/200 Abcam) and monoclonal alkaline phosphatase-conjugated sheep anti-DIG (1/1000, Roche) antibodies at 4°C overnight. The alkaline phosphatase was revealed by the Fast red (546 nm, Sigma) reaction and anti-MVH antibody by goat anti-rabbit (Alexa 488, Molecular probe). Finally, the nuclei were stained with DAPI and the signals were detected using an Sp5 bi-photon microscope.

Polyadenylated mRNAs were detected by *in situ* hybridization on tubule squash preparations, using a biotinylated DNA oligo dT probe. Cells were incubated overnight in 70% ethanol, rehydrated for 15 min in 2× SSC/ 15% formamide. Hybridization of probe (10 ng) was performed in 30 µl hybridization solution [2× SSC/ 15% formamide/ 10% dextran sulfate/ 0.5 µg/mL yeast tRNA/ 4 mM VRC] at 37°C overnight. Cells were washed twice for 20 min. in 2× SSC/ 15% formamide and for 10 min. in SSC 1×. Oligo-dT probes were detected using TRITC-labelled streptavidin during the primary antibody hybridization step Immunodetection of the CB, using anti-MVH antibody, was performed as described above.

### Sucrose density gradient fractionation

Purified germ cells from P23, P28 and P40 testes were resuspended in 50 mM Tris-HCl pH 7.5, / 50 mM KCl / 10 mM MgAc/ 200 U/mL RNasin/ 100 µg/mL cycloheximide/ 1 mM DTT buffer and lysed by vortexing with glass beads (Sigma). Extracts were loaded onto continuous 15–50% sucrose gradients prepared in lysis buffer without detergent. Gradients were centrifuged for 2 h 45′ at 39 000 rpm in an SW41 rotor (Beckman Coulter). Fractions (1 mL) were collected using a Foxy collector (Isco) and the absorbance was recorded at A_260_. RNA was purified with Trizol (GIBCO BRL) from 2/3 of each fraction and analysed by RT-PCR. Proteins were precipitated from the remaining 1/3 and analysed by Western blot. The presence of CB proteins in the post-nuclear supernatant was verified by Western blot analysis using supernatant (1/40) and pellet (membranes/nuclei) (1/100) extracts.

### Protein/mRNA co-immunoprecipitation, semi-quantitative RT-PCR and Western blot analysis

Protein and RNA immunoprecipitation (IP) was performed as described in [62] with some modifications. Briefly, proteins from 20.10^6^ spermatogenic cells were extracted in 400 µl lysis buffer (10 mM Tris-HCl, pH 7.5, 10 mM NaCl, 2 mM EDTA, pH 8, 0.5% Triton X-100, protease inhibitor cocktail). After centrifugation (10 000 g, 20 min, 4°C), the pellet was discarded. One fifth (100 µl) of the cytoplasmic extract was kept for RNA extraction and the rest (400 µl) was precleared for 1 h at 4°C in 50 µl washed protein A Sepharose (Sigma) supplemented with 10 µl RNAsin; it was then divided into 2 aliquots which were incubated overnight at 4°C in 50 µl of washed protein A-sepharose coated with 15 µg of 3A2 or mouse IgG1 for HuR IP and 2 µg of anti-MVH (Abcam) or rabbit IgG for MVH IP. Unbound extract (100 µl out of 400 µl) was kept for RNA extraction; beads were washed 8 times with 500 µl Net-2 buffer. RNAs in the bound, unbound and cytoplasmic fractions were extracted using Trizol After extraction, 1/5 of the RNA was used for reverse transcription; the presence of candidate target mRNAs was tested by semi-quantitative RT-PCR. Conditions and primers are available upon request.

Protein extraction and Western-blot analysis were performed as previously described [20], [63] using monoclonal anti-HuR 3A2 antibodies (1/1000; gift of I. Gallouzi), monoclonal anti-alpha tubulin (1/1000; Sigma), polyclonal anti-MVH (1/200; gift of N. Kotaja and P. Sassone-Corsi), polyclonal anti-S6 (1/1000, Cell Signaling), anti-AUF1 (UpState Biotechnology) and anti-Dcp1 (1/500) (generous gift of B. Seraphin).

### Protein/protein co-immunoprecipitation

HuR and MVH proteins were immunoprecipitated as described in “Protein/mRNA co-immunoprecipitation” with some modifications: after IP, Protein A Sepharose beads were washed only 3 times. The column-bound proteins were denatured in Laemmli buffer/DTT at 95°C and analyzed by Western blot using HuR and MVH antibodies. For RNAse treatment, samples were supplemented with 2 µg RNAse A after IP and incubated for 10 min at 30°C.

## Supporting Information

Figure S1 HuR localization in the CB of spermatids was confirmed by in situ hybridization performed on (non-staged) tubule squash preparations, using oligo dT (red) combined with immunofluorescence, using anti-HuR antibody (green). CBs are indicated by arrows. Scale bar: 5 µm.
(0.25 MB TIF)

Figure S2 Heat map showing Brd2 and GCNF mRNA expression in germ cells and 19 somatic tissues (Chalmel et al. 2007 and supplementary Table 2). B-Alignment of 3′ UTR of GCNF and Brd2 mRNAs reveals the conservation of ARE (red bold characters) and binding site for miR181a/c. Accession numbers for GCNF: NM_010264.3 (Mus musculus), NM_033334.2 (Homo sapiens), XM_342427.3 (Rattus norvegicus), XM_001500647.2| (Equus caballus) and XM_001235477.1 (Gallus gallus) ; for Brd2: NM_010238.3 (Mus musculus), NM_005104.2, (Homo sapiens), NM_212495.1 (Rattus norvegicus). C. Germ cell cytoplasmic extracts from a pool of P23 or P40 testes were fractionated on 15–50% sucrose density gradients (Rib.Sub.U corresponds to the dedimentation of small, large ribosomal sub unit as well as monosomes). RNAs were extracted and expression of GCNF mRNAs was analyzed by semi-quantitative RT-PCR, using primers specific for the longest transcript (L) or recognizing both transcripts (L+S). GCNF L is the predominant GCNF mRNA in pachytene spermatocytes, whereas GCNF S is expressed predominantly in haploid round spermatids (Yang et al. 2003). While GCNF L transcripts shift from polysomes to mRNPs between P23 and P40, interpretation of the results for GCNF S is less obvious. It would require the use of GCNF S-specific primers, such as GSP-T18 (described in [29]), which unfortunately gave unspecific signals when used on RNAs extracted from gradients. The graphs represent the percentage of GCNF (L+S or L) signal in a given fraction, expressed relative to the sum of the intensities found in the 10 fractions, considered as 100%.
(0.15 MB TIF)

Figure S3 In situ hybridization and immunohistochemistry were performed on dried down slides from adult testis to study Brd2 mRNAs (anti-sense probe: red) and HuR (green) colocalization in the CB, besides their principal expression in the nucleus. Merge pictures show their localization in the CB (yellow+arrows) of early round spermatids. Scale bar: 5 µm.
(0.35 MB TIF)

Table S1 Information about the mouse and human microarray samples
(0.07 MB XLS)

Table S2 Murine and human ARE-mRNAs were extracted from the ARED Organism database (left column). Their level of expression was analyzed using a mouse and a human expression dataset as described in [19]. The mouse samples include one murine testicular somatic cell type (Sertoli cells), three male germ cell types (spermatogonia, pachytene spermatocytes and round spermatids), seminiferous tubules and whole testis, as well as 19 normal non-testicular tissue types. The human samples include two male germ cell types (pachytene spermatocytes, round spermatids), seminiferous tubules, as well as 19 healthy non-testicular tissue types [19] (Table S1).
(1.14 MB XLS)
