# Supplementary figures and images for: Temporally Regulated Traffic of HuR and Its Associated ARE-Containing mRNAs from the Chromatoid Body to Polysomes during Mouse Spermatogenesis
Source: PLoS One. 2009 Mar 31;4(3):e4900. doi: 10.1371/journal.pone.0004900 (PMC2659425; doi:10.1371/journal.pone.0004900)

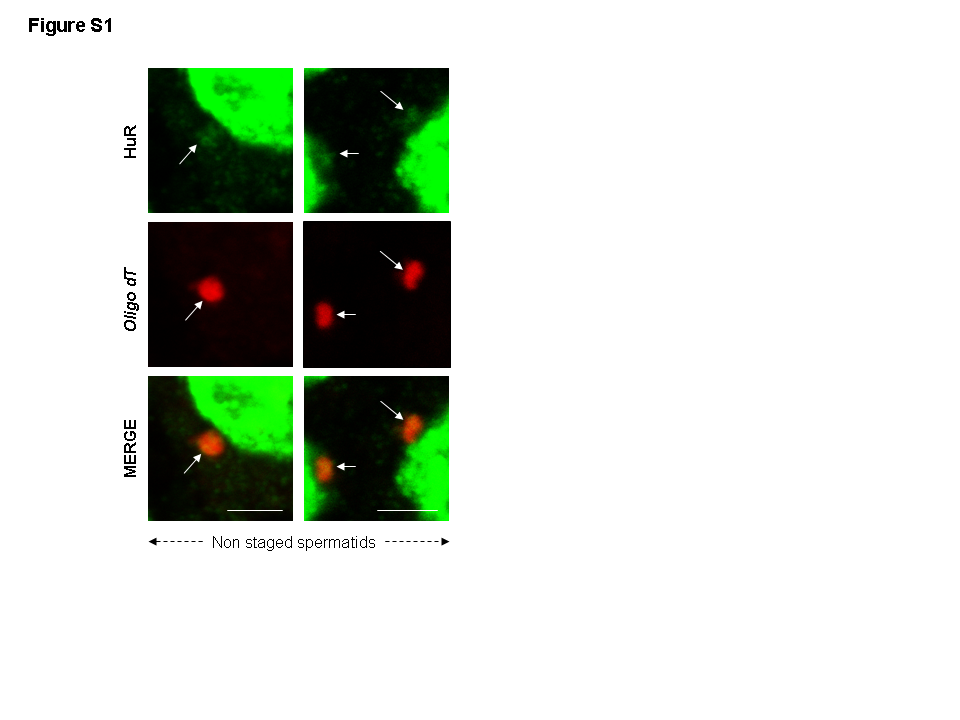

Supplement: Figure S1 — HuR localization in the CB of spermatids was confirmed by in situ hybridization performed on (non-staged) tubule squash preparations, using oligo dT (red) combined with immunofluorescence, using anti-HuR antibody (green). CBs are indicated by arrows. Scale bar: 5 µm. (0.25 MB TIF) [file pone.0004900.s001.tif]

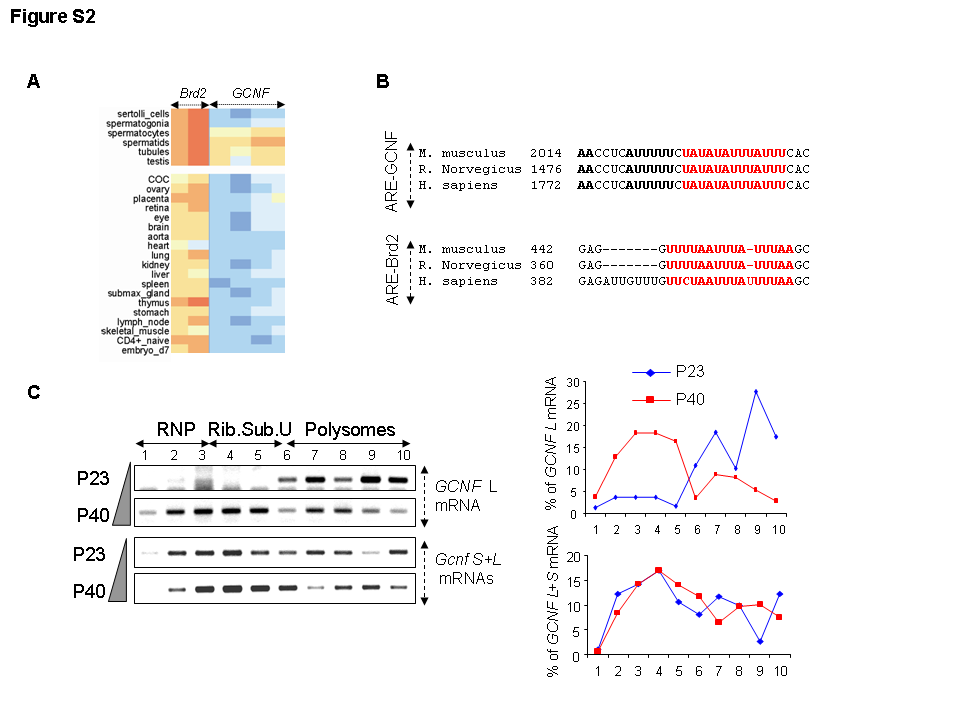

Supplement: Figure S2 — Heat map showing Brd2 and GCNF mRNA expression in germ cells and 19 somatic tissues (Chalmel et al. 2007 and supplementary Table 2). B-Alignment of 3′ UTR of GCNF and Brd2 mRNAs reveals the conservation of ARE (red bold characters) and binding site for miR181a/c. Accession numbers for GCNF: NM_010264.3 (Mus musculus), NM_033334.2 (Homo sapiens), XM_342427.3 (Rattus norvegicus), XM_001500647.2| (Equus caballus) and XM_001235477.1 (Gallus gallus) ; for Brd2: NM_010238.3 (Mus musculus), NM_005104.2, (Homo sapiens), NM_212495.1 (Rattus norvegicus). C. Germ cell cytoplasmic extracts from a pool of P23 or P40 testes were fractionated on 15–50% sucrose density gradients (Rib.Sub.U corresponds to the dedimentation of small, large ribosomal sub unit as well as monosomes). RNAs were extracted and expression of GCNF mRNAs was analyzed by semi-quantitative RT-PCR, using primers specific for the longest transcript (L) or recognizing both transcripts (L+S). GCNF L is the predominant GCNF mRNA in pachytene spermatocytes, whereas GCNF S is expressed predominantly in haploid round spermatids (Yang et al. 2003). While GCNF L transcripts shift from polysomes to mRNPs between P23 and P40, interpretation of the results for GCNF S is less obvious. It would require the use of GCNF S-specific primers, such as GSP-T18 (described in [29]), which unfortunately gave unspecific signals when used on RNAs extracted from gradients. The graphs represent the percentage of GCNF (L+S or L) signal in a given fraction, expressed relative to the sum of the intensities found in the 10 fractions, considered as 100%. (0.15 MB TIF) [file pone.0004900.s002.tif]

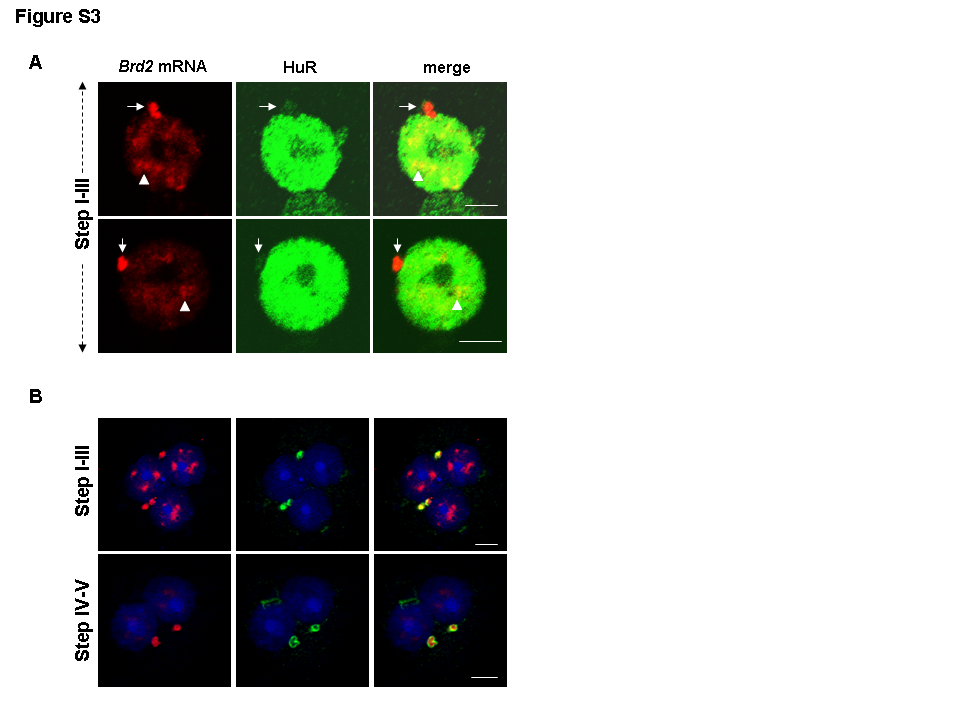

Supplement: Figure S3 — In situ hybridization and immunohistochemistry were performed on dried down slides from adult testis to study Brd2 mRNAs (anti-sense probe: red) and HuR (green) colocalization in the CB, besides their principal expression in the nucleus. Merge pictures show their localization in the CB (yellow+arrows) of early round spermatids. Scale bar: 5 µm. (0.35 MB TIF) [file pone.0004900.s003.tif]
